# Shocking Revelations and Saccharin Sweetness in the Study of *Drosophila* Olfactory Memory

**DOI:** 10.1016/j.cub.2013.07.060

**Published:** 2013-09-09

**Authors:** Emmanuel Perisse, Christopher Burke, Wolf Huetteroth, Scott Waddell

**Affiliations:** 1 Centre for Neural Circuits and Behaviour, The University of Oxford, Tinsley Building, Mansfield Road, Oxford, OX1 3SR, United Kingdom; 2 Department of Neurobiology, University of Massachusetts Medical School, 364 Plantation Street, Worcester, MA 01605, USA

## Abstract

It is now almost forty years since the first description of learning in the fruit fly *Drosophila melanogaster*. Various incarnations of the classic mutagenesis approach envisaged in the early days have provided around one hundred learning defective mutant fly strains. Recent technological advances permit temporal control of neural function in the behaving fly. These approaches have radically changed experiments in the field and have provided a neural circuit perspective of memory formation, consolidation and retrieval. Combining neural perturbations with more classical mutant intervention allows investigators to interrogate the molecular and cellular processes of memory within the defined neural circuits. Here, we summarize some of the progress made in the last ten years that indicates a remarkable conservation of the neural mechanisms of memory formation between flies and mammals. We emphasize that considering an ethologically-relevant viewpoint might provide additional experimental power in studies of *Drosophila* memory.

## Main Text

### Introduction

Much of our cellular understanding of memory has come from the study of relatively simple invertebrate preparations, such as *Aplysia* [1], or from deciphering mechanisms of synaptic plasticity in isolated mammalian brain slices [2]. Early studies of olfactory memory initiated in the fruit fly *Drosophila melanogaster* promised to harness the power of classical genetics to uncover the mechanics of learning in the fly brain [3]. The first mutants and transgenic studies revealed an involvement of the cyclic adenosine monophosphate (cAMP) signaling pathway [4–10]. However, it was very difficult to isolate the affected genes and thus to transition from mutant fly to mechanistic insight. A variety of recent technical advances have now enabled precise, temporally controlled neural manipulations [11–16] and physiological recordings [17,18]. These additional lines of investigation, taken with the considerable recent advances in our understanding of the neural processing of olfaction, have invigorated the field and provided some genuinely novel insight. *Drosophila* now is a realistic model nervous system in which to study how memories are acquired, stored and retrieved within the context of functioning neural circuits.

Here, we summarize findings over the last 10 years from studies of olfactory memory that we find of particular interest and that illustrate the changing approaches and the progress made in defining the underlying neural mechanisms. In addition, we highlight the utility of considering an ethological perspective when studying fly learning. Other, non-olfactory fly memory paradigms were recently reviewed [19,20].

### Behavioral Assays

The first studies of olfactory learning in *Drosophila* employed variants of an aversive paradigm pairing an odor with an electric shock [3,21]. This was soon followed by an appetitive paradigm pairing odors with sugar rewards (Box 1) [22]. Regardless of the training procedure, groups of flies are tested for memory using a binary odor choice in a T-maze.

It seems reasonable to consider how these laboratory paradigms relate to the behavior of flies in the wild. Electric shock is believed to signal pain but is certainly not a natural stimulus that flies routinely encounter. In contrast, the association between an odor and a source of food is likely to be important for foraging behavior. However, it is harder to control ingestion of sugar than switching on shock delivery. Formation of aversive long-term memory (LTM) requires multiple training trials with intervening rest intervals [23], whereas appetitive LTM is rapidly formed in a single session. This difference alone is likely to reflect the evolutionary importance of food-seeking for the fly. Although the speed of LTM formation was recently described in detail [24,25], it was evident in the perdurance of memory in the first study [22]. In fact, most of the underlying phenomena that drive current investigation were noted decades ago in flies, or other animals [26–28]. However, technical advances now provide the opportunity to understand the phenomena, at the level of detailed neural circuitry and molecules mediating functional connectivity.

The considerable progress made using the current population-based memory paradigms suggests that they are well suited to answering many of the key questions in the field. However, they are not infallible. Flies in the wild do not usually march in swarms through plastic tubes and in most assays, learning indices normalize differences in individual performance. Normalization can be useful to iron out unexplained variation but it is critical to know whether the flies behave as individuals in the memory assay, or whether they influence each other’s decisions. This was addressed in the very first study of fly learning [3]. Two groups of flies that were trained differently were mixed before testing and were shown to independently choose the relevant odor of the two presented [3]. Nevertheless, others have recently reported that interactions between trained flies can occur in certain situations [29,30] so it remains something important to consider.

Furthermore, there is no doubt in our minds that variation between individual flies is real. Most fly behaviorists who study individuals are aware of it and differences in olfactory-driven behaviors are visible in data where individual flies are analyzed. Furthermore, two recent studies detail plausible contributing neural-based reasons for individual differences [31,32] — part of the olfactory system is randomly wired [31] and stochastic transposition in neural genomes [32] leads to genetic heterogeneity in the brain. Such variability may ultimately prove to be beyond investigator control and a better understanding of the individual fly will therefore be essential. Assays of olfactory choice in free-flying individuals are feasible [33] but are not currently *en vogue*.

### Monitoring and Manipulating Memory Circuits

Combined with robust learning assays, a primary strength of *Drosophila* as a model comes from the ability to alter the activity of the nervous system with cellular precision. State-of-the-art genetic tools provide fly trainers with an impressive level of neural control. Key amongst those are transgenic flies with gene expression limited to restricted populations of neurons. There are now three independent binary transcription systems — GAL4–UAS [34], LexA–lexAop [35] and QF–QUAS [36] — that permit expression of a variety of label and effector transgenes (see below). In principle, by selecting the appropriate transgenic lines from the very large publically accessible collections [37], it is now possible to simultaneously control three distinct populations of cells in the brain of an intact, freely moving fly.

A variety of effector transgenes serve to dissect the role of the identified neurons. One of the earliest developed tools that revolutionized the field was the dominant-negative temperature-sensitive dynamin transgene cloned from the paralytic *Shibire*^ts1^ mutant fly [14]. Simply elevating the temperature of flies >29°C reversibly blocks synaptic transmission from neurons expressing UAS-*shibire*^ts1^ (UAS-*shi*^ts1^). The acute requirement of output from these neurons can then be investigated.

Optogenetic neural control with either the purinoreceptor P2X2 [15] or Channelrhodopsin [38,39] can be used to activate specific neurons in a behaving fly through light stimulation. Stimulating defined neurons in the brain allows one to ask, without any prior knowledge of how the neurons are activated, whether they have a causal role in the generation of specific behavioral patterns. This approach provides reproducibility and cellular resolution to the age-old and historically valuable technique of electrical brain stimulation [40–42]. However, due to experimental ease and cost, most fly memory investigators have stimulated neurons using expression of the temperature-controlled Transient Receptor Potential (TRP) channel dTrpA1 [16], and to a lesser extent TRPM8 [43] transgenes. dTrpA1 conducts Ca^2+^ and depolarizes neurons that express it, when flies are exposed to temperatures >25°C [16]. Therefore, the same temperature regimes used to block transmission from specific neurons with *shibire* [14] can be used to stimulate neurons with UAS-*dTrpA1* [16].

Ultimately, one needs to know how the identified neurons connect to each other. Resolving the precise neural anatomy of individual neurons can be difficult because even restricted GAL4 lines often label neurons with intertwined processes. Recent fly versions of the mouse Brainbow allow for a somewhat random labeling of neighboring neurons in unique colors by using recombination to switch the fluorescent protein transgene that is available to be driven by GAL4 [44,45]. Alternatively, single neurons within GAL4-expressing populations can be visualized in a more directed way using photoactivatable/convertible variants of GFP [46,47]. Expressing any combination of a number of neural compartment markers fused to fluorescent proteins or epitope tags can indicate neural polarity [48–52].

Knowing which branches of a particular neuron are potentially transmitting or receiving signals allows one to use GFP reconstituted across synaptic partners (GRASP) to assess whether the putative pre- and post-synaptic compartments of distinct neurons of interest are close enough to potentially form functional synapses [53,54].

Another way to assess functional connectivity is to remove the appropriate neurotransmitter receptors from presumed postsynaptic partner neurons by transgenic RNAi [55]. If, for example, one has identified an instructive octopamine-releasing neuron, removing octopamine receptors from the relevant downstream neurons should abolish the gain-of-function effects of stimulating the octopaminergic neuron [56]. This neural circuit epistasis approach permits one to assemble functional neural circuits.

To fully understand circuit function, however, there is little substitute for synaptic physiology. Genetically encoded tools have also had a significant impact on this area. One can stimulate presynaptic neurons with opto- or thermogenetic control while recording postsynaptically with the fluorescent calcium indicator GCaMP [18,57] or using electrophysiology. Although they lack the temporal resolution and sensitivity of electrophysiology, the most recent and highly sensitive versions of GCaMP [18] allow recording of neural ensemble activity with relative ease. This enviable collection of approaches means that *Drosophila* memory experiments are mostly constrained by imagination and creativity.

### Olfactory Memory Acquisition

#### Representing Odors in the Brain

**Figure 1:**
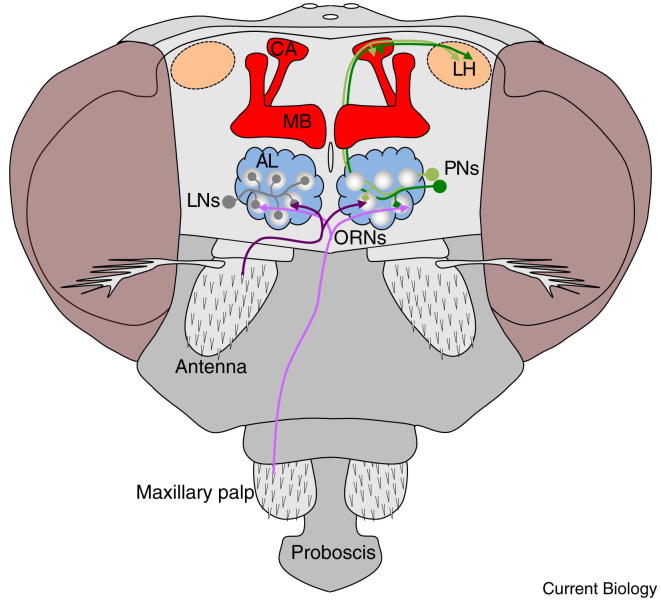
The *Drosophila* olfactory pathway. Olfactory receptor neurons (ORNs) in sensillae on the 3^rd^ antennal segments and the maxillary palps project their axons bilaterally into individual glomeruli in the antennal lobe (AL). In these glomeruli, ORN input is integrated and processed by the action of mostly multiglomerular excitatory and inhibitory local interneurons (LNs). Processed odor information is then relayed to the calyx (CA) of the mushroom body (MB) and the lateral horn (LH) by uniglomerular projection neurons (PNs).

Flies detect air-borne odors using olfactory receptor neurons (ORNs) housed in hair-like sensilla on their maxillary palps and antennae. ORNs expressing the same odorant receptor genes project their axons to two bilateral symmetrical glomeruli in the antennal lobes (Figure 1) [58]. This organization suggests that odors are represented as combinatorial patterns of activity in antennal lobe glomeruli. However, a considerable number of studies have now demonstrated that the initial odor code is richer than activation or inhibition of ORNs. Electrophysiological recordings of ORNs reveal a diverse range of odor-evoked temporal patterns [59,60] and behavioral experiments suggest these temporal signals provide ample information for the fly to discriminate odors [61]. In addition, dendrites of ORNs are grouped in pairs, triplets or quartets in sensilla and recent work has shown that activation of one ORN can interfere with the activity of their neighbors within the same sensillum via a process called ephaptic signaling [62]. Therefore, each ORN is unlikely to represent an isolated input and it may be more accurate to think of sensillar ORN clusters as interactive groups.

In the antennal lobe, ORNs form synapses with projection neurons, as well as local inhibitory and excitatory interneurons (Figure 1) [63–66]. This elaborate network, which includes many gap junctions, provides a level of gain control, maintaining odor representations consistent over a range of odor concentrations [66–69]. The prevailing projection neuron type has dendrites in a single glomerulus [70], suggesting a faithful transmission of odor information from ORNs to projection neurons. However, networks of local interneurons in the antennal lobe transform incoming ORN inputs into a more broad and temporally rich signal across the projection neuron ensemble [64–66,71,72].

Odor information carried by projection neurons is delivered to the mushroom body calyx and the lateral horn (Figure 1), as well as back into the antennal lobe. At present it is largely believed that the ∼2000 neurons of the mushroom body encode learned responses to odors [73] whereas the lateral horn processes innate responses [74]. Projection-neuron arbors in the lateral horn appear to at least be segregated between projection neurons handling fruit odors and those for pheromones [75].

**Figure 2:**
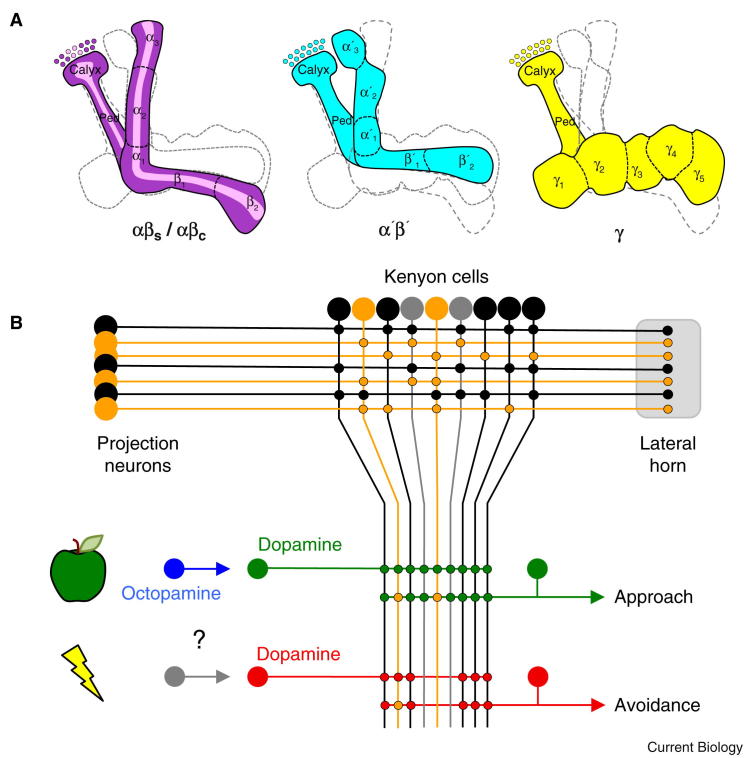
Olfactory memories are formed in mushroom body Kenyon cells. (A) Illustration of four functional classes of Kenyon cells in the mushroom body. Each Kenyon cell has a dendrite in the calyx and a long neurite that projects down the peduncle and into the lobes. Kenyon cells that contribute to the αβ (purple/pink) or α′β′ (cyan) division bifurcate and send one axon branch into the vertical α or α′ lobe and one to the horizontal β or β′ lobe, respectively. In contrast γ Kenyon cells (yellow) send a single projection in the horizontal γ lobe. The ∼1000 αβ neurons can be further subdivided into the surface (αβ_s_, purple) and core (αβ_c_, pink). There are ∼400 α′β′ and ∼700 γ neuron. At least 15 non-overlapping zones have been defined along the lobes (α_1-3_, β_1,2_, α′_1-3_, β′_1,2_, and γ_1-5_) based on the innervation of input and output neurons (Figures 3 and 5). (B) Basic circuit model of olfactory memory. Odors are represented as projection neuron driven activation (orange) of sparse populations of mushroom body Kenyon cells in each functional subdivision (black and grey cells). Downstream neurons guiding conditioned approach or avoidance pool Kenyon cell outputs. Food presentation during appetitive conditioning potentiates the odor activated Kenyon cell output synapses onto approach relevant neurons. Sweetness of sugar reinforces memory via the action of octopamine neurons working through rewarding dopamine neurons that directly innervate specific zones within the mushroom body lobes. Nutrient content of sugar activates the rewarding dopamine neurons via an unknown pathway. Conversely, aversive conditioning strengthens odor activated Kenyon cell output synapses onto avoidance relevant neurons via the action of other negative dopamine neurons that directly innervate distinct zones within the mushroom body lobes.

Physiological recordings suggested that projection neurons are randomly connected to mushroom body Kenyon cells [76,77] and this has recently been confirmed using photoconvertible GFP and electroporation to label 200 individual Kenyon cells and their presynaptic projection neurons, respectively [31]. Tracing each projection neuron back to the antennal lobe glomerulus it innervates showed that each subtype of Kenyon cell — αβ, α′β′ and γ (Figure 2A) — integrates projection neuron input from different and apparently random glomeruli (Figure 2B). Given this connectivity, it seems likely that odors will be represented differently in the mushroom body of each individual fly. Nevertheless, it may not matter which particular projection neurons transmit the odor information, or which particular Kenyon cells represent it, as long as each odor is represented as a sparse pattern of activity across every functional subclass of Kenyon cell. Recent functional imaging using a more sensitive GCaMP variant revealed a larger number of Kenyon cells that were odor-activated [78] than in previous studies [79]. However, responses to monomolecular and complex odors remained consistently odor-specific and patterns were relatively sparse within the overall population of Kenyon cells [78].

Broadly tuned projection neuron activity is converted into sparse Kenyon cell responses. In the locust (*Schistocerca americana*), this is achieved by a combination of mechanisms: Kenyon cells detect coincidence of projection neuron inputs, Kenyon cells display a sub-threshold oscillatory activity and broadly innervating neurons provide inhibitory feedback [80,81]. Oscillations have not yet been observed in *Drosophila* Kenyon cells. However, Kenyon cell anatomy suggests that coincidence detection is likely. An individual Kenyon cell has an average of seven dendritic claws, with each claw receiving one projection neuron synapse [31,76,77,82]. Therefore, each Kenyon cell could integrate inputs from seven or more projection neurons. In the locust, giant GABA-ergic neurons densely innervate the calyx, proximal peduncle and the α-lobe of the mushroom body and refine odor signals in Kenyon cells [81,83]. The giant GABA-ergic neurons are non-spiking and are thus likely to provide local microcircuit inhibition. The *Drosophila* equivalent of the giant GABA-ergic neurons are believed to be the GABA-ergic Anterior Paired Lateral (APL) neurons [83] which ramify throughout the entire mushroom body neuropil [84,85], including the calyx where they could interact with Kenyon cell dendrites and projection neurons. The APL neurons (Figure 4A) have recently been shown to limit odor-evoked activity in Kenyon cells [86]. Furthermore, the observation that blocking APLs’ inhibitory influence enhances learning [85,87] could reflect a broader activation of Kenyon cells by odor. Dendrites of αβ and γ but not α′β′ Kenyon cells in the mushroom body calyx also have presynaptic compartments [88]. It is not yet clear what these putative recurrent connections do, but they could potentially amplify odor-evoked Kenyon cell responses and/or recruit lateral inhibition.

#### Reinforcement

Reinforcement pathways add value to odor-activated Kenyon cell synapses. For several years the dogma was that the aversive value of electric shock was assigned to odor-activated Kenyon cell synapses by dopamine, whereas octopamine (the invertebrate equivalent of noradrenaline) signaled reward [73,89]. However, recent work has demonstrated that for 10 years the field was misled by the limited expression of a tyrosine hydroxylase (TH) promoter fragment GAL4 line used to manipulate dopamine neurons [56,90]. It is now apparent that octopamine, dopamine and serotonin all shape the reinforcement signals and that distinct populations of mushroom body-innervating dopamine neurons ultimately provide both aversive and appetitive reinforcement for olfactory memory (Figure 2B) [56,90–95].

#### Negative Reinforcement

**Figure 3:**
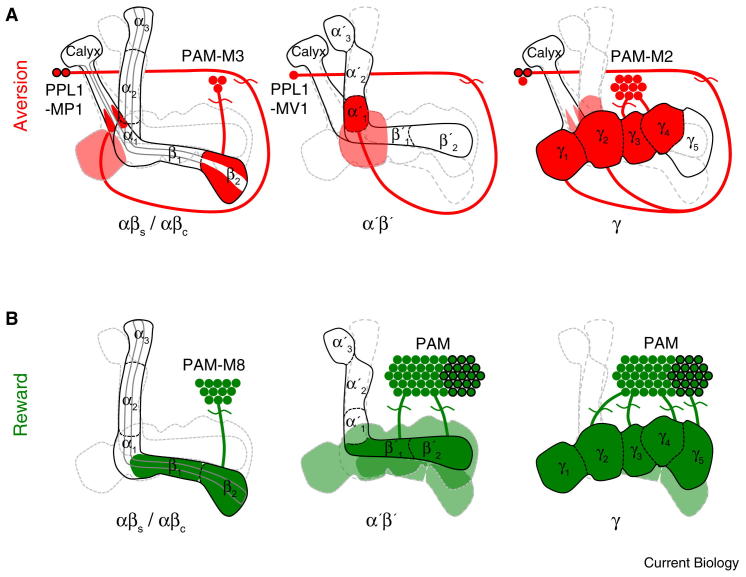
The dopaminergic reinforcement system. (A) Aversive reinforcing dopamine neurons (red) reside in the PPL1 cluster lateral to the calyx and the PAM cluster in front of the horizontal lobes. The PPL1 neurons MP1 and MV1 innervate the γ_1_ (heel) and the distal αβ surface of the peduncle, or the γ_2_ (junction) and α′_1_ regions, respectively. Both MP1 and MV1 receive input in the anterior superior median protocerebrum. The PAM cluster cell types M2 and M3 also convey aversive reinforcement. M2 innervates γ_3_ and γ_4,_ whereas M3 innervates the β_2_ surface. They receive input in the crepine region near the horizontal MB lobes. (B) Rewarding dopamine neurons (green) are located in the PAM cluster. PAM contains ∼140 dopamine neurons that ramify throughout the horizontal mushroom body lobes but the full extent of the rewarding dopamine cells is unclear. Separate input regions in the posterior superior lateral protocerebrum and crepine imply functional subdivision. Octopamine delivers the reinforcing effects of sweet taste through the OAMB receptor expressed in a subset of PAM dopamine cells (black outline). OA simultaneously exerts an essential modulatory effect through OCTβ2R in the aversive reinforcing MP1 neurons. In (A) and (B) each panel emphasizes innervation of the relevant dopaminergic neurons within the αβ, α′β′ or γ lobe.

The first study to establish a role for dopaminergic neurotransmission during memory acquisition concluded a specific effect for aversive learning [89]. Blocking TH–GAL4 expressing neurons with UAS-*shi*^ts1^ [14] impaired electric-shock reinforced olfactory learning but left sucrose reinforced memory [89]. The dopamine neurons responsible for aversive reinforcement were mapped using artificial activation. Optogenetic activation of TH–GAL4 neurons expressing P2X2 during odor presentation formed aversive memory in single flies [91]. Aversive memories were formed when activating TH–GAL4 neurons but not with a GAL4 line that lacked expression in a cluster of dopaminergic neurons called Protocerebral Posterior Lateral 1 (PPL1). Photo-activatable GFP demonstrated that mushroom body-innervating dopaminergic neurons in the PPL1 cluster innervate the vertical mushroom body lobe (Figure 3A).

Studies using dTrpA1-mediated activation established that two classes of PPL1 dopamine neurons and two from another cluster, called Protocerebral Anterior Medial (PAM), can convey negative reinforcement [93]. These negatively reinforcing dopamine neurons project to discrete and non-overlapping regions of the αβ, α′β′ and γ lobes of the mushroom body, suggesting that aversive reinforcement might be distributed across the major mushroom body subdivisions [91–93]. Furthermore, experiments with shock-reinforcement indicated these dopamine neurons reinforce memories of differing strength and persistence, implying a plausible combinatorial effect of dopamine neurons in the formation of aversive memory [92,93]. However, genetic restoration of the dDA1 dopamine receptor exclusively in the γ neurons was sufficient to reinforce short- and long-term aversive memory, suggesting that the most critical aversive signals are mediated through the Kenyon cells in the γ lobe [96]. It will be important to test this model with other negative reinforcing stimuli, such as bad taste or nausea [97]. Although shock plausibly activates nociceptors in the fly, the relevant neural pathways linking shock sensation to dopamine neuron activation are not known.

#### Positive Reinforcement

Sucrose has historically been used as the unconditioned stimulus in *Drosophila* appetitive conditioning [22]. However, using sugars of differing dietary utility revealed that sweet taste and nutritional value contribute differently to appetitive memory formation [98,99]. Sweet sugars with no nutritional value, such as arabinose, reinforced robust short-term memory (STM) whereas sweet nutritious sugars, such as sucrose and fructose, formed robust appetitive LTM [98]. Surprisingly, nutrient reward is processed quickly. Flies taught to discriminate between an odor paired with a sweet and nutritious compound and an odor paired with a sugar that is just sweet prefer the nutrient-coupled odor within two minutes of training [98]. This makes some sense, because foraging flies feed on rotting fruits rich in nutritious sucrose and fructose. It seems logical for a foraging animal to be able to rapidly assign nutrient information to a food source and to remember it.

Importantly, these studies with different naturally occurring sugars suggested that parallel pathways reinforce appetitive memory and that a post-ingestive nutrient signal provides input to the long-term reinforcement circuitry [98]. Octopamine neurons represent the reinforcing effects of sweet taste. Blocking them using Tdc2–GAL4 (*Tdc2* encodes tyrosine decarboxylase, an enzyme required for octopamine production) and UAS-*shi*^ts1^ impaired conditioning with sweet non-nutritious arabinose but did not disrupt learning with sweet and nutritious sucrose. Furthermore, dTrpA1-mediated stimulation of octopamine neurons formed memory that did not persist, like that formed with an only sweet sugar [56]. Therefore, nutrient signals required for LTM reinforcement are independent of octopamine neurons. Analyzing subsets of octopamine neurons that innervate the mushroom body revealed that a more distributed octopamine signal was required for appetitive memory formation. But which circuits does octopamine control?

A study of flies mutant for the dopamine receptor dDA1 revealed a defect in aversive and appetitive olfactory learning [100]. Subsequently, the PAM cluster of dopamine neurons that is not labeled in TH-GAL4 flies was found to contain a population of rewarding neurons. dTrpA1-mediated stimulation of PAM dopamine neurons while presenting odor forms appetitive olfactory memory [56,90] that requires the dDA1 receptor expressed in the mushroom body [90]. Furthermore, blocking PAM dopamine neurons during training with UAS-*shi*^ts1^ impaired formation of both sweetness and nutrient-reinforced memory [56,90]. Reward-encoding PAM dopamine neurons project exclusively to the horizontal mushroom body lobes (Figure 3B) [56,90]. Lastly, *in vivo* Ca^2+^ imaging in PAM dopamine neurons revealed that rewarding dopamine neurons respond when flies are fed sucrose [90].

Octopamine provides key input to the rewarding dopamine neurons [56]. Octopamine neuron activation paired with odor could not form appetitive memory in flies that were mutant for the dDA1 receptor. In contrast, stimulating PAM dopamine neurons formed robust appetitive memory in *Tbh* mutant flies that lack octopamine, demonstrating that rewarding dopamine neurons are functionally downstream of octopamine [56].

Octopamine-dependent memory requires the α-adrenergic like receptor OAMB in rewarding dopamine neurons (Figure 3B). Reducing *oamb* expression with transgenic RNAi impaired arabinose-reinforced but not sucrose-reinforced learning, consistent with the model that octopamine mediates sweet taste but not nutrient reinforcement [56]. The direct octopamine-dopamine neuron link was further strengthened with GRASP and GCaMP live-imaging of an increase in intracellular Ca^2+^ in rewarding dopamine neurons when octopamine was applied to the exposed brain [56].

Octopamine-dependent memory also requires the β-adrenergic-like receptor OCTβ2R. Surprisingly, *octβ2R* was only required to implant memory in satiated but not hungry flies, indicating an interaction between octopamine and neurons representing motivational control [56]. Further experiments demonstrated that expression of *octβ2R* in, and transmission from, the aversive MB-MP1 dopamine neurons is required for octopamine-implanted memory [56].

Unlike blocking octopamine neurons, blocking the rewarding dopamine neurons during training also impaired nutritious sucrose-conditioned appetitive memory [56,90]. It is not clear which dopamine neurons provide the essential reward signals and where they project to within the mushroom bodies. The PAM cluster has more than 120 neurons and those that are able to reinforce appetitive memory innervate multiple non-overlapping zones on the β, β′ and γ lobes [56,90] (Figure 3B). This organization suggests a very different process from those orchestrated by the ca. three aversive-reinforcing neurons in the PPL1 cluster. It will be interesting to understand what the increased numerical complexity provides. Manipulating the activity of subsets of rewarding PAM dopamine neurons and restoring dDA1 expression to subsets of mushroom body neurons [96] might help in this regard.

It is currently unclear how a nutrient signal is conveyed to rewarding dopamine neurons. It could in principle involve identified neural circuits and receptors that regulate sugar feeding behavior [101,102]. Nevertheless, the available data [56,90] support a new model for rewarding reinforcement in *Drosophila*. The sweet taste of sugar engages a distributed octopamine signal that activates rewarding dopamine neurons through OAMB while simultaneously modulating MB-MP1 dopamine neurons (Figure 3A) through OCTβ2R. In addition rewarding dopamine neurons integrate post-ingestive nutrient signals, if present, to reinforce appetitive LTM. These rewarding dopamine neurons drive synaptic plasticity at odor-activated Kenyon cell synapses and engage the consolidation process.

### Memory Storage

Once formed, memories are either consolidated and remembered for the long-term, or forgotten. Both of these processes appear to involve active neural processes after training in the fly. The consolidation process was first revealed as a time-dependent disruption of memory with cold-anesthesia delivered after training [103]. Cold-anesthesia renders the animals inactive, and it is therefore assumed that it disrupts neural signaling. In addition, experiments using other disruptive agents suggest the existence of two forms of consolidated memory [23,104]. These forms have been confusingly named anesthesia-resistant memory (ARM) and long-term memory (LTM). The features that distinguish ARM from LTM are only evident with aversive memory — sequential trials without intervening rest (massed training) can lead to ARM whereas multiple trials with intervening rest (spaced training) lead to LTM [23]. Furthermore, LTM requires new protein synthesis after training and is consequently sensitive to cycloheximide feeding, whereas ARM is less sensitive to blockers of protein synthesis [23]. Experiments with appetitive conditioning suggest a different relationship and less clear distinction between ARM and LTM [24]. Nevertheless, the difference between massed and spaced trained aversive memory formation [23] inspires many investigations in the field and is often of considerable interest from the perspective of psychology and learning theory [105].

Some of the neural circuitry that maintains labile memory and consolidates longer-lasting forms of memory is known. Analysis of the forgetful *amnesiac* mutant flies uncovered the importance of the Dorsal Paired Medial (DPM) neurons [106]. Expressing UAS-*shi*^ts1^ in DPM neurons revealed that transmission from DPM neurons is dispensable during training but is essential after training to consolidate aversive and appetitive memory [24,107–109]. Although the *amnesiac* gene was originally reported to encode a putative neuropeptide [110], and several studies indicate the importance of *amnesiac* expression in DPM neurons [106,108,109,111,112], the function of the encoded peptide remains unclear. A recent report suggests that DPM neurons release serotonin (5-HT) and that 5-HT is required for ARM formation [113].

**Figure 4:**
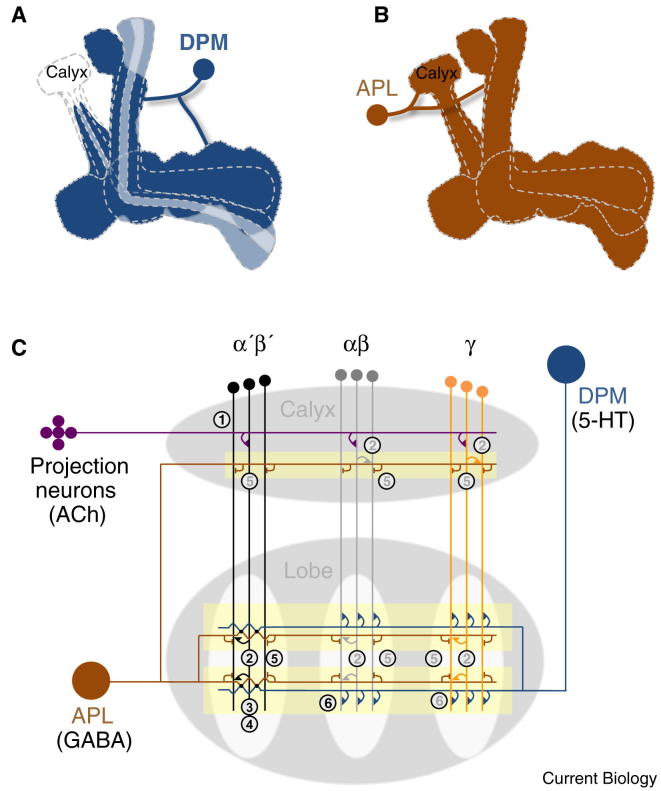
Circuitry of consolidation. (A) Output from DPM neurons after training is required for memory consolidation. DPM neurons ramify throughout the mushroom body, except the calyx. Innervation of the α′β′ and γ lobe appears more dense than in the αβ lobe, and the αβ_c_ in particular, but the functional significance is not known. Expression of the GFP-tagged presynaptic marker protein bruchpilot predominantly localized to the αβ lobes, implying plausible functional directionality of DPM from the α′β′ and γ lobes to the αβ lobe. (B) Output from APL neurons is required after training to maintain labile memory. Inhibitory APL neurons innervate the entire mushroom body neuropil and show no obvious regional preference. APL neurons are gap junction-coupled to DPM neurons. (C) Model for memory consolidation. Cholinergic (ACh) projection neurons transfer olfactory information to αβ, α′β′ and γ Kenyon cell dendrites in the mushroom body calyx (1). Within microcircuit domains (shaded yellow) the odor-activated and learning-potentiated Kenyon cells activate the APL neuron (2). The APL depolarizes the DPM neuron through gap junctions mainly in the α′β′ lobes (3). The serotonergic (5-HT) DPM closes a reverberant circuit loop by releasing transmitter onto Kenyon cells (4). APL neurons provide lateral inhibition within the mushroom body lobes and calyx, maintaining signal specificity within the recurrent network (5). Over time, DPM-released 5-HT consolidates memory in the αβ Kenyon cells through the d5HT1A receptor (6).

Each DPM neuron innervates all the lobes and the base of the peduncle of the ipsilateral mushroom body [106] (Figure 4A). Functional imaging of odor-evoked activity in DPM neurons, and the localization of neural compartment markers, suggests that the DPM neurons are pre- and postsynaptic to mushroom body neurons [108,114]. In addition, odor-evoked DPM neuron activity has been useful to report on memory processing in the mushroom body. Odor-evoked signals are increased in DPM neurons following presentation of odor and electric-shock under the microscope [108]. Interestingly, this elevated signal is evident 30 min after odor-shock pairing and persists for at least 1 hour, a time window during which DPM transmission is required for memory consolidation [107,108].

Imaging odor-evoked DPM responses after appetitive conditioning [112] revealed a temporal dynamic that nicely reflects the unique nature of appetitive memory reinforcement [56,98] and the established role for DPM neurons in appetitive memory consolidation [24]. The conditioned odor evoked an elevated response in DPM neurons for more than twice as long (2.5 h) following training with nutritious substances than with non-nutritive sugar (about 1 h) [112]. Furthermore, whereas the non-nutritive or shock reinforced traces were only evident in the vertical branch of the DPM neuron projections into the mushroom body, the nutrient-prolonged trace was observable in both the vertical and horizontal DPM projections [112]. The persistent appetitive trace in the horizontal DPM branch/lobes coincides with the innervation of the positively reinforcing dopamine neurons [56,90] and it is plausible that these and other dopamine neurons control the consolidation process [115,116]. However, it is not clear why the non-nutritive sugar memory, which also requires rewarding dopamine neurons, would form a trace in the vertical DPM branch.

Transmission from the α′β′ neurons, but not from αβ or γ, is required at a similar time as the activity of DPM neurons [24,117], which suggests a recurrent α′β′-DPM circuit loop that maintains activity and drives appetitive and aversive memory consolidation after training [118]. Modeled recurrent networks are intrinsically unstable without an inhibitory component [119]. Remarkably, dye injection into DPM neurons revealed that DPM neurons are gap junction-coupled to the GABA-ergic APL neurons (Figure 4A) [120]. APL neurons are, therefore, an integral part of the DPM-α′β′ circuit and are likely to be concurrently activated with DPM neurons, although the gap junctions may be directional or rectified. Blocking APL neurons with UAS-*shi*^ts1^ after appetitive conditioning revealed that their transmission is required for early memory but not for LTM consolidation [114]. In addition, compromising expression of the relevant gap junction-forming innexins in either DPM or APL disrupted non-consolidated aversive memory [120]. Whereas GRASP suggests DPM contacts mushroom body neurons in all of the lobes, APL appears to contact mushroom body neurons in the α′β′ lobes, but makes little direct contact with those in the distal α lobe [114]. It was proposed that the APL neurons provide widespread inhibition to stabilize labile memory traces held in a recurrent DPM-α′β′-APL network while memory consolidation proceeds in the αβ neurons driven by DPM processes (Figure 4B) [114].

The involvement of the mushroom body neurons in memory processing may be more complex than the established αβ, α′β′ and γ, subdivision. Blocking the output of all of the αβ neurons, or subdivisions within αβ, for extended periods after training disrupted appetitive and aversive LTM [121].

### Forgetting

In principle, memories can be eliminated or disrupted by simple decay, by reversal of consolidation, or by interference with memories of opposing experience. Recent studies in *Drosophila* suggest that the processes of consolidation and forgetting may be functionally interwoven. Using GCaMP imaging, reinforcing dopamine neurons were found to exhibit rhythmic oscillatory activity after aversive training and this persistent network activity appears to inhibit ARM and promote LTM formation [115]. Importantly, blocking aversive reinforcing dopamine neurons enhanced ARM. Another study [116] confirmed that aversive dopamine neurons oscillate after training but concluded that dopamine neuron activity is required to forget labile appetitive and aversive memories. In this study, blocking aversive reinforcing dopamine neurons after training enhanced the persistence of labile non-consolidated memory. In addition, flies mutant for the DAMB dopamine receptor showed enhanced memory stability, suggesting that signals through this receptor after training may regulate an active forgetting process. Consistent with such a model, both studies found that stimulating reinforcing dopamine neurons (perhaps akin to unpaired reinforcement delivery [122]) after training weakened memory [115,116].

The Rac1 GTPase pathway is also involved in fly forgetting. Inhibiting Rac1 activity throughout the nervous system slowed memory decay and converted aversive memories usually lasting a few hours into those persisting for a day. Furthermore, elevating Rac1 expression in the mushroom body accelerated aversive memory decay [123]. Although it is currently unclear which signals regulate the Rac1 pathway to promote forgetting, Rac1 apparently interacts with radish [124,125], which is critical for aversive ARM [124] and appetitive LTM [24]. As appetitive memories can be intrinsically more stable than aversive memories [22], it will be interesting to test the role of rewarding dopamine neurons after training and DAMB and Rac1 manipulations in appetitive conditioning.

### Memory Retrieval

Memories must be available for recall at any time. Reminiscent of mammals, several studies have now shown that in *Drosophila* there is a time-dependent reorganization of the circuits driving memory-guided behavior after training [24,96,117,121,126,127]. The *shi*^ts1^ transgene [14] has been the tool of choice in most of these studies and for good reason. It seems reasonable that a requirement for synaptic output is a prerequisite for claiming a role of particular neurons in retrieval. However, as blocking transmission anywhere along the odor-activated pathway from olfactory sensory neurons to motor output could in principle result in a retrieval effect, care is required. One needs to know that the flies still recognize the odors, can discriminate between the odors, and can move appropriately. In addition, one might imagine that *Drosophila* has several circuits that when acutely altered might distract from the intended task.

Before summarizing the findings it is worth noting that investigators frequently use different assays, GAL4 drivers and unique time points and temporal manipulations. We are therefore piecing this together with these inconsistencies in mind. Furthermore, it should by this point in the review be apparent that there are many differences between shock-reinforced aversive memory and appetitive memory reinforced with sugar. Therefore, although both memories are olfactory, one should not assume that the underlying neural and molecular mechanisms of memory will be identical between the two paradigms.

STM is usually measured minutes to an hour after training and the current consensus is that it relies on the mushroom body γ neurons. Restoring DopR or the *rutabaga*-encoded Type I adenylate cyclase to mushroom body γ neurons of *DopR* or *rut* mutant flies, respectively, rescued aversive STM [11,96,126,128]. Therefore, key reinforcement signals for aversive STM are likely to be delivered to the γ neurons. However, a strong requirement for γ neuron output during STM retrieval is not evident [129,130].

Appetitive memory exhibits a more definitive role for neurotransmission from γ neurons [127]. Restoring *rutabaga* expression to mushroom body αβ or γ neurons of *rut* mutant flies rescued 2h memory. In addition, temporal block with UAS-*shi*^ts1^ or constitutive block with tetanus toxin of γ neurons impaired immediate and 2h appetitive memory, whereas similar block of αβ neurons lacked consequence. The authors concluded that parallel memories are formed in αβ and γ but that the γ trace dictates early behavioral performance [127].

**Figure 5:**
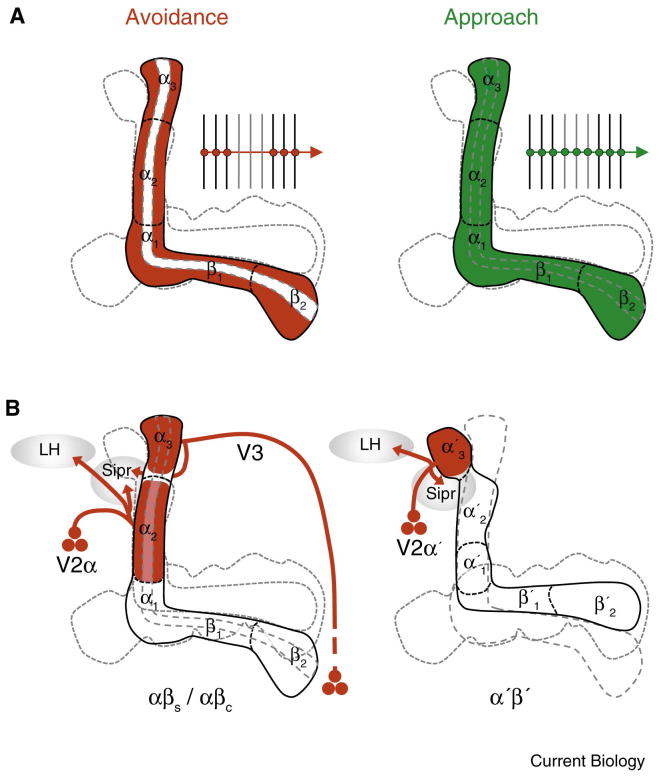
Circuitry of retrieval. (A) Aversive and appetitive memories are driven by different populations of mushroom body Kenyon cells. Aversive memory requires output from αβ_s_ but not αβ_c_ neurons. In contrast, appetitive memory requires output from αβ_s_ and αβ_c_ neurons. This distinct requirement implies differential pooling of αβ outputs by relevant output neurons (represented in the insets and in Figure 2B). (B) V2 and V3 output neurons are required for aversive memory retrieval. The approximately seventy V2 neurons fall into at least two morphological categories. V2α and V2α′ neurons have dendrites in the α lobe surface and α′ lobe, respectively. Dendrites of the V3 neurons innervate the tip of the α lobe. V2α, V2α′ and V3 neurons all project outputs to the superior intermediate protocerebrum (sipr) and the lateral horn (LH).

As stated above, output from the α′β′ mushroom body neurons is required after training to consolidate memory [24,117]. The field typically refers to the three hour time point as middle-term memory (MTM). However, it is not totally clear if this point reflects a unique phase or a transition between STM and LTM. Notably, output is required from the αβ neurons at this time [117,129,131,132]. For aversive and appetitive memory retrieval work suggests that the αβ neurons are of particular importance for LTM. Output from the αβ neurons but not the γ neurons is required for aversive [132] and appetitive LTM [127] and an aversive LTM memory trace can be detected from 9–24 h in αβ neurons [133,134]. Output from the αβ posterior [135] but not from the αβ core neurons [121] is required for an aversive LTM retrieval, suggesting a role for αβ subdivision. In fact, recent work has shown that αβ core neuron output is specifically required for the retrieval of consolidated appetitive memory [136] (Figure 5A). Olfactory memories of differing value are therefore represented in distinct αβ neuron populations. This differential arrangement seems to be generated by stratified innervation of aversive and rewarding dopaminergic neurons within the αβ lobes (Figure 3). Although no requirement has been demonstrated for γ neuron output in LTM retrieval, a later aversive LTM trace has been reported in γ neurons from 18 to 48 h after training [134]. Therefore, distinct populations of mushroom body neurons contribute to memory retrieval, with the role for αβ neurons becoming more critical as time progresses after training.

### Mushroom Body Output Pathways

Odor memories are believed to be represented as modifications in synaptic weights between Kenyon cells and mushroom body output neurons [73,137]. Hence, defining the neural pathways that are postsynaptic to Kenyon cells is a major area of interest so that one can measure potential learning-related changes in synaptic efficacy. In addition, one might expect that a time-dependent alteration in the mushroom body neurons that drive learned behavior will be observable as a time-dependent requirement for different output neurons that are postsynaptic to the relevant mushroom body neuron subtypes.

The MB-V2 output neurons (Figure 5B) are required for aversive STM and LTM retrieval [138], suggesting they provide necessary drive for conditioned avoidance. In addition, odor-evoked responses observed in MB-V2 neurons are reduced after aversive conditioning, which indicates that the connection between the mushroom body Kenyon cells and MB-V2 neurons may be modified by learning. Interestingly, output from MB-V2 neurons is dispensable for appetitive STM retrieval [138]. This makes some sense because MB-V2α neurons have dendrites that are restricted to the periphery of the α-lobe stalk where the αβ surface neurons reside (Figure 5B) [136,138]. It therefore appears that MB-V2α neurons do not pool the αβ core neuron outputs that are critical for appetitive memory retrieval [136]. The MB-V2α′ neurons have dendrites in the tip of the α′ lobe [138] but their function in retrieval has not been clearly demonstrated.

A requirement for output from MB-V3 neurons has also been reported for aversive LTM retrieval (Figure 5) [135]. MB-V3 neuron dendrites innervate the tip of the α-lobe. Blocking protein synthesis in the MB-V3 neurons impaired LTM [135]. Since MB-V3 are postsynaptic to Kenyon cells, this could reflect a postsynaptic component of LTM. Aversive LTM formation can proceed when dopamine receptors are only provided in the mushroom body [96], so presynaptic dopamine receptor activation is able to drive the necessary plasticity. A role for MB-V3 neurons in appetitive memory has not been reported.

A role for new protein synthesis was also suggested for the Dorsal Anterior Lateral (DAL) neurons and blocking output from DAL neurons disrupted aversive LTM retrieval [139]. However, the anatomical relationship of DAL neuron connectivity to mushroom body neurons makes a role for DAL less easy to comprehend than for the MB-V3 neurons [135]. DAL neurons are putatively connected to heat-sensitive neurons [140] and the dendritic region of the αβ posterior Kenyon cells in the accessory calyx [139]. DAL may therefore be presynaptic to αβ posterior Kenyon cells that do not receive direct olfactory input from projection neurons. Without an obvious link to odor-activated Kenyon cells, it is currently unclear how odor-specific consolidated memory could be represented, or influenced, by the DAL neurons. Interestingly, appetitive LTM does not rely on the DAL neurons [141].

Retrieved memories need to be able to guide behavioral choices at the appropriate time. Sugar-reinforced appetitive memory formation and expression exhibits a clear dependence on the satiation state of the fly. Memories are only formed and robustly expressed if the fly is hungry [22,24,142]. The motivational state dependence of memory formation may simply reflect the need to ingest reinforcing sucrose, since appetitive memories can be formed independent of state by stimulating the relevant rewarding octopamine or dopamine neurons [56,90]. However, expression of learned behavior is acutely controlled by a hierarchical inhibitory mechanism. Experiments suggest that in hungry flies Neuropeptide F (dNPF), the fly ortholog of mammalian NPY, disinhibits the MB-MP1 dopamine neurons to permit appetitive memory retrieval [142]. Interestingly, these dopamine neurons are those that have also been implicated in aversive reinforcement and memory consolidation/forgetting [93,115,116]. It will, therefore, be critical to understand how these potential functions relate. Live-imaging the MB-MP1 neurons suggests their oscillatory activity is suppressed by hunger [143] consistent with their proposed role in motivational control of appetitive memory retrieval [142]. It will be important to understand the physiological mechanism through which MB-MP1 neurons gate appetitive memory retrieval.

### Outlook

The future is bright for the budding fly memory researcher. A great deal of hard work has been done to establish assays, isolate a considerable number of memory-defective mutant flies, devise sophisticated genetic approaches and a neural circuit framework. Most of the mutants have only been superficially studied. Therefore, the time has now come to put some of these gene products into the neural circuit perspective and to understand why each mutant fly is ‘dumb’. It will be of similar interest to understand how reinforcement signals, neural networks and intracellular signaling cascades contribute to the generation of physiological memory traces and how each trace and signaling pathway relates to the next.

Finding that both aversive and rewarding reinforcement and motivation is mediated by dopamine brings the known functions of the fly dopamine system closer to those of mammals [94]. This indicates a profound conservation of neural mechanism but there are still several interesting differences. For example, studies in mammals [144], and in the pond snail [145], suggest an exclusive role for dopamine in the consolidation of appetitive memory and for noradrenaline, or octopamine, in the consolidation of aversive memory. It would be worthwhile to test whether the adrenergic modulation of dopaminergic neurons that was revealed in appetitive learning in the fly [56] is a conserved feature, and also whether octopamine through dopamine plays a role in fly aversive memory consolidation.

Understanding how the same dopamine signal generates memories of opposite value [56,90–93], regulates consolidation [115] and forgetting [116] and provides motivational control [142] is another key question. Part of that explanation appears to be due to downstream signaling [116]. The rest could be anatomical with distinct dopamine neurons conveying different meaning to unique zones of the same mushroom body neurons [94]. The elaborate organization of dopaminergic neurons and that of the neural circuits of memory consolidation brings a pressing need for synaptic physiology at defined junctions. In addition, it will be critical to investigate signal transduction cascades with the resolution of cellular compartments.

## Box 1 Glossary.

**Anesthesia-resistant memory:** a consolidated *Drosophila* memory developing after training that is resistant to anesthetic disruption and is dependent on the *radish* gene.

**Appetitive memory:** a memory whose expression involves approach behavior. In the fly experiments discussed here, this involves training flies by pairing one odorant with sucrose reward and another with nothing. When subsequently given the choice between the two odorants in a T-maze, the flies preferentially approach the previously sugar-paired odorant. Appetitive learning and memory performance are facilitated by hunger and suppressed by satiety and are therefore considered to be goal-directed actions.

**Aversive memory:** a memory whose expression involves avoidance behavior. In the fly experiments discussed here this involves training flies by pairing one odorant with electric shock and another with nothing. When subsequently given the choice between the two odorants in a T-maze the flies preferentially avoid the previously shock-paired odorant. A new automated version of the assay also allows trial-and-error learning where single flies sample two odor streams and learn to avoid the odor stream triggering punishment [91].

**Binary transcription system:** a genetic system for controlling the induction of gene expression. An activator line that expresses the transcriptional activators *GAL4* (from a yeast), *LexA* (from a bacterium) or *QF* (from a fungus) genes under the control of a tissue-specific promoter is crossed to an effector line that carries the DNA-binding motif of GAL4/LexA/QF (upstream activating sequence, UAS/LexA operator/QUAS) fused to the gene of interest. As a result, the progeny of this cross express the gene of interest in a cell-specific manner.

**Calyx:** a compartment of the mushroom bodies where the presynaptic projection neurons synapse with the dendrites of the Kenyon cells.

**Consolidation:** memory consolidation refers to the progressive post-acquisition stabilization of long-term memory, as well as to the memory phase(s) during which such presumed stabilization takes place.

**Glomeruli**: morphologically distinguishable areas in the antennal lobe that contain the presynaptic terminals of olfactory receptor neurons that express the same olfactory receptor, local inhibitory and excitatory interneuron connections and dendrites of postsynaptic projection neurons.

**Kenyon cells:** the intrinsic neurons comprising the mushroom bodies.

**Lateral horn:** an area defined by the terminal arborizations of projection neurons in the lateral protocerebrum.

**Learning index or Performance index:** a numerical index of memory performance in the T-maze assay. It is calculated as the number of flies in the ‘correct’ arm of the maze minus those in the ‘wrong’ arm of the maze divided by the total number of flies in the experiment.

**Long-term memory:** consolidated memory that requires new protein synthesis after training.

**Middle-term memory:** a phase of fly memory that lasts a few hours, is sensitive to anesthetic disruption and is dependent on the *amnesiac* gene.

**Mushroom bodies:** mushroom bodies, or *Corpora pedunculata*, are paired neural structures in the insect brain that morphologically resemble mushrooms and are required for olfactory memory.

**Reinforcement:** depending on the context, reinforcement is either a rewarding or aversive stimulus or the neural representation of the reinforcing stimulus.

**Short-term memory:** a phase of fly memory that lasts a few minutes and is sensitive to anesthetic disruption.

## References

[1] Kandel E.R. (2001). The molecular biology of memory storage: a dialogue between genes and synapses. Science.

[2] Nicoll R.A., Roche K.W. (2013). Long-term potentiation: Peeling the onion. Neuropharmacology.

[3] Quinn W.G., Harris W.A., Benzer S. (1974). Conditioned behavior in Drosophila melanogaster. Proc. Natl. Acad. Sci. USA.

[4] Dudai Y., Jan Y.N., Byers D., Quinn W.G., Benzer S. (1976). dunce, a mutant of Drosophila deficient in learning. Proc. Natl. Acad. Sci. USA.

[5] Byers D., Davis R.L., Kiger J.A.J. (1981). Defect in cyclic AMP phosphodiesterase due to the dunce mutation of learning in Drosophila melanogaster. Nature.

[6] Chen C.N., Denome S., Davis R.L. (1986). Molecular analysis of cDNA clones and the corresponding genomic coding sequences of the Drosophila dunce+ gene, the structural gene for cAMP phosphodiesterase. Proc. Natl. Acad. Sci. USA.

[7] Livingstone M.S., Sziber P.P., Quinn W.G. (1984). Loss of calcium/calmodulin responsiveness in adenylate cyclase of rutabaga, a Drosophila learning mutant. Cell.

[8] Levin L.R., Han P.L., Hwang P.M., Feinstein P.G., Davis R.L., Reed R.R. (1992). The Drosophila learning and memory gene rutabaga encodes a Ca2+/Calmodulin-responsive adenylyl cyclase. Cell.

[9] Drain P., Folkers E., Quinn W.G. (1991). cAMP-dependent protein kinase and the disruption of learning in transgenic flies. Neuron.

[10] Yin J.C., Wallach J.S., Del V.M., Wilder E.L., Zhou H., Quinn W.G., Tully T. (1994). Induction of a dominant negative CREB transgene specifically blocks long-term memory in Drosophila. Cell.

[11] McGuire S.E., Le P.T., Osborn A.J., Matsumoto K., Davis R.L. (2003). Spatiotemporal rescue of memory dysfunction in Drosophila. Science.

[12] Gohl D.M., Silies M.A., Gao X.J., Bhalerao S., Luongo F.J., Lin C.C., Potter C.J., Clandinin T.R. (2011). A versatile in vivo system for directed dissection of gene expression patterns. Nat. Methods.

[13] Pfeiffer B.D., Jenett A., Hammonds A.S., Ngo T.T., Misra S., Murphy C., Scully A., Carlson J.W., Wan K.H., Laverty T.R. (2008). Tools for neuroanatomy and neurogenetics in Drosophila. Proc. Natl. Acad. Sci. USA.

[14] Kitamoto T. (2001). Conditional modification of behavior in Drosophila by targeted expression of a temperature-sensitive shibire allele in defined neurons. J. Neurobiol..

[15] Lima S.Q., Miesenbock G. (2005). Remote control of behavior through genetically targeted photostimulation of neurons. Cell.

[16] Hamada F.N., Rosenzweig M., Kang K., Pulver S.R., Ghezzi A., Jegla T.J., Garrity P.A. (2008). An internal thermal sensor controlling temperature preference in Drosophila. Nature.

[17] Miesenbock G., De Angelis D.A., Rothman J.E. (1998). Visualizing secretion and synaptic transmission with pH-sensitive green fluorescent proteins. Nature.

[18] Akerboom J., Chen T.W., Wardill T.J., Tian L., Marvin J.S., Mutlu S., Calderon N.C., Esposti F., Borghuis B.G., Sun X.R. (2012). Optimization of a GCaMP calcium indicator for neural activity imaging. J. Neurosci..

[19] Pitman J.L., DasGupta S., Krashes M.J., Leung B., Perrat P.N., Waddell S. (2009). There are many ways to train a fly. Fly (Austin).

[20] Kahsai L., Zars T. (2011). Learning and memory in Drosophila: behavior, genetics, and neural systems. Int. Rev. Neurobiol..

[21] Tully T., Quinn W.G. (1985). Classical conditioning and retention in normal and mutant Drosophila melanogaster. J. Comp. Physiol. [A].

[22] Tempel B.L., Bonini N., Dawson D.R., Quinn W.G. (1983). Reward learning in normal and mutant Drosophila. Proc. Natl. Acad. Sci. USA.

[23] Tully T., Preat T., Boynton S.C., Del V.M. (1994). Genetic dissection of consolidated memory in Drosophila. Cell.

[24] Krashes M.J., Waddell S. (2008). Rapid consolidation to a radish and protein synthesis-dependent long-term memory after single-session appetitive olfactory conditioning in Drosophila. J. Neurosci..

[25] Colomb J., Kaiser L., Chabaud M.A., Preat T. (2009). Parametric and genetic analysis of Drosophila appetitive long-term memory and sugar motivation. Genes Brain Behav..

[26] Tolman E.C. (1932). Purposive Behavior in Animals and Men.

[27] Pavlov I.V., Gantt H. (1928). Lectures on Conditioned Reflexes: The Higher Nervous Activity of Animals. Trans..

[28] Dethier V.G. (1976). The Hungry Fly - a Physiological Study of the Behaviour Associated with Feeding.

[29] Chabaud M.A., Isabel G., Kaiser L., Preat T. (2009). Social facilitation of long-lasting memory retrieval in Drosophila. Curr. Biol..

[30] Chabaud M.A., Preat T., Kaiser L. (2010). Behavioral characterization of individual olfactory memory retrieval in Drosophila melanogaster. Front Behav. Neurosci..

[31] Perrat P.N., DasGupta S., Wang J., Theurkauf W., Weng Z., Rosbash M., Waddell S. (2013). Transposition-driven genomic heterogeneity in the Drosophila brain. Science.

[32] Caron S.J., Ruta V., Abbott L.F., Axel R. (2013). Random convergence of olfactory inputs in the Drosophila mushroom body. Nature.

[33] Frye M.A., Tarsitano M., Dickinson M.H. (2003). Odor localization requires visual feedback during free flight in Drosophila melanogaster. J. Exp. Biol..

[34] Brand A.H., Perrimon N. (1993). Targeted gene expression as a means of altering cell fates and generating dominant phenotypes. Development.

[35] Lai S.L., Lee T. (2006). Genetic mosaic with dual binary transcriptional systems in Drosophila. Nat. Neurosci..

[36] Potter C.J., Tasic B., Russler E.V., Liang L., Luo L. (2010). The Q system: a repressible binary system for transgene expression, lineage tracing, and mosaic analysis. Cell.

[37] Jenett A., Rubin G.M., Ngo T.T., Shepherd D., Murphy C., Dionne H., Pfeiffer B.D., Cavallaro A., Hall D., Jeter J. (2012). A GAL4-Driver Line Resource for Drosophila Neurobiology. Cell Rep..

[38] Boyden E.S., Zhang F., Bamberg E., Nagel G., Deisseroth K. (2005). Millisecond-timescale, genetically targeted optical control of neural activity. Nat. Neurosci..

[39] Schroll C., Riemensperger T., Bucher D., Ehmer J., Voller T., Erbguth K., Gerber B., Hendel T., Nagel G., Buchner E. (2006). Light-induced activation of distinct modulatory neurons triggers appetitive or aversive learning in Drosophila larvae. Curr. Biol..

[40] Hess W., Bonin G.V. (1964). The Biology of the Mind. Trans..

[41] Delgado J.M.R. (1969). Physical Control of the Mind: Toward a Psychocivilized Society.

[42] Valenstein E.S. (1973). Brain Stimulation and Motivation: Research and Commentary.

[43] Peabody N.C., Pohl J.B., Diao F., Vreede A.P., Sandstrom D.J., Wang H., Zelensky P.K., White B.H. (2009). Characterization of the decision network for wing expansion in Drosophila using targeted expression of the TRPM8 channel. J. Neurosci..

[44] Hadjieconomou D., Rotkopf S., Alexandre C., Bell D.M., Dickson B.J., Salecker I. (2011). Flybow: genetic multicolor cell labeling for neural circuit analysis in Drosophila melanogaster. Nat. Methods.

[45] Hampel S., Chung P., McKellar C.E., Hall D., Looger L.L., Simpson J.H. (2011). Drosophila Brainbow: a recombinase-based fluorescence labeling technique to subdivide neural expression patterns. Nat. Methods.

[46] Datta S.R., Vasconcelos M.L., Ruta V., Luo S., Wong A., Demir E., Flores J., Balonze K., Dickson B.J., Axel R. (2008). The Drosophila pheromone cVA activates a sexually dimorphic neural circuit. Nature.

[47] Ruta V., Datta S.R., Vasconcelos M.L., Freeland J., Looger L.L., Axel R. (2010). A dimorphic pheromone circuit in Drosophila from sensory input to descending output. Nature.

[48] Estes P.S., Ho G.L., Narayanan R., Ramaswami M. (2000). Synaptic localization and restricted diffusion of a Drosophila neuronal synaptobrevin–green fluorescent protein chimera in vivo. J. Neurogenet..

[49] Wang J., Ma X., Yang J.S., Zheng X., Zugates C.T., Lee C.H., Lee T. (2004). Transmembrane/juxtamembrane domain-dependent Dscam distribution and function during mushroom body neuronal morphogenesis. Neuron.

[50] Wagh D.A., Rasse T.M., Asan E., Hofbauer A., Schwenkert I., Durrbeck H., Buchner S., Dabauvalle M.C., Schmidt M., Qin G. (2006). Bruchpilot, a protein with homology to ELKS/CAST, is required for structural integrity and function of synaptic active zones in Drosophila. Neuron.

[51] Nicolai L.J., Ramaekers A., Raemaekers T., Drozdzecki A., Mauss A.S., Yan J., Landgraf M., Annaert W., Hassan B.A. (2010). Genetically encoded dendritic marker sheds light on neuronal connectivity in Drosophila. Proc. Natl. Acad. Sci. USA.

[52] Owald D., Fouquet W., Schmidt M., Wichmann C., Mertel S., Depner H., Christiansen F., Zube C., Quentin C., Korner J. (2010). A Syd-1 homologue regulates pre- and postsynaptic maturation in Drosophila. J. Cell Biol..

[53] Feinberg E.H., Vanhoven M.K., Bendesky A., Wang G., Fetter R.D., Shen K., Bargmann C.I. (2008). GFP Reconstitution Across Synaptic Partners (GRASP) defines cell contacts and synapses in living nervous systems. Neuron.

[54] Gordon M.D., Scott K. (2009). Motor control in a Drosophila taste circuit. Neuron.

[55] Dietzl G., Chen D., Schnorrer F., Su K.C., Barinova Y., Fellner M., Gasser B., Kinsey K., Oppel S., Scheiblauer S. (2007). A genome-wide transgenic RNAi library for conditional gene inactivation in Drosophila. Nature.

[56] Burke C.J., Huetteroth W., Owald D., Perisse E., Krashes M.J., Das G., Gohl D., Silies M., Certel S., Waddell S. (2012). Layered reward signalling through octopamine and dopamine in Drosophila. Nature.

[57] Nakai J., Ohkura M., Imoto K. (2001). A high signal-to-noise Ca(2+) probe composed of a single green fluorescent protein. Nat. Biotechnol..

[58] Laissue P.P., Vosshall L.B. (2008). The olfactory sensory map in Drosophila. Adv. Exp. Med. Biol..

[59] de Bruyne M., Foster K., Carlson J.R. (2001). Odor coding in the Drosophila antenna. Neuron.

[60] Hallem E.A., Ho M.G., Carlson J.R. (2004). The molecular basis of odor coding in the Drosophila antenna. Cell.

[61] DasGupta S., Waddell S. (2008). Learned odor discrimination in Drosophila without combinatorial odor maps in the antennal lobe. Curr. Biol..

[62] Su C.Y., Menuz K., Reisert J., Carlson J.R. (2012). Non-synaptic inhibition between grouped neurons in an olfactory circuit. Nature.

[63] Wilson R.I., Laurent G. (2005). Role of GABAergic inhibition in shaping odor-evoked spatiotemporal patterns in the Drosophila antennal lobe. J. Neurosci..

[64] Shang Y., Claridge-Chang A., Sjulson L., Pypaert M., Miesenbock G. (2007). Excitatory local circuits and their implications for olfactory processing in the fly antennal lobe. Cell.

[65] Olsen S.R., Bhandawat V., Wilson R.I. (2007). Excitatory interactions between olfactory processing channels in the Drosophila antennal lobe. Neuron.

[66] Olsen S.R., Wilson R.I. (2008). Lateral presynaptic inhibition mediates gain control in an olfactory circuit. Nature.

[67] Root C.M., Masuyama K., Green D.S., Enell L.E., Nassel D.R., Lee C.H., Wang J.W. (2008). A presynaptic gain control mechanism fine-tunes olfactory behavior. Neuron.

[68] Ignell R., Root C.M., Birse R.T., Wang J.W., Nassel D.R., Winther A.M. (2009). Presynaptic peptidergic modulation of olfactory receptor neurons in Drosophila. Proc. Natl. Acad. Sci. USA.

[69] Yaksi E., Wilson R.I. (2010). Electrical coupling between olfactory glomeruli. Neuron.

[70] Wong A.M., Wang J.W., Axel R. (2002). Spatial representation of the glomerular map in the Drosophila protocerebrum. Cell.

[71] Wilson R.I., Turner G.C., Laurent G. (2004). Transformation of olfactory representations in the Drosophila antennal lobe. Science.

[72] Bhandawat V., Olsen S.R., Gouwens N.W., Schlief M.L., Wilson R.I. (2007). Sensory processing in the Drosophila antennal lobe increases reliability and separability of ensemble odor representations. Nat. Neurosci..

[73] Heisenberg M. (2003). Mushroom body memoir: from maps to models. Nat. Rev. Neurosci..

[74] Heimbeck G., Bugnon V., Gendre N., Keller A., Stocker R.F. (2001). A central neural circuit for experience-independent olfactory and courtship behavior in Drosophila melanogaster. Proc. Natl. Acad. Sci. USA.

[75] Jefferis G.S., Potter C.J., Chan A.M., Marin E.C., Rohlfing T., Maurer C.R.J., Luo L. (2007). Comprehensive maps of Drosophila higher olfactory centers: spatially segregated fruit and pheromone representation. Cell.

[76] Turner G.C., Bazhenov M., Laurent G. (2008). Olfactory representations by Drosophila mushroom body neurons. J. Neurophysiol..

[77] Murthy M., Fiete I., Laurent G. (2008). Testing odor response stereotypy in the Drosophila mushroom body. Neuron.

[78] Honegger K.S., Campbell R.A., Turner G.C. (2011). Cellular-resolution population imaging reveals robust sparse coding in the Drosophila mushroom body. J. Neurosci..

[79] Wang Y., Guo H.F., Pologruto T.A., Hannan F., Hakker I., Svoboda K., Zhong Y. (2004). Stereotyped odor-evoked activity in the mushroom body of Drosophila revealed by green fluorescent protein-based Ca2+ imaging. J. Neurosci..

[80] Perez-Orive J., Bazhenov M., Laurent G. (2004). Intrinsic and circuit properties favor coincidence detection for decoding oscillatory input. J. Neurosci..

[81] Gupta N., Stopfer M. (2012). Functional analysis of a higher olfactory center, the lateral horn. J. Neurosci..

[82] Leiss F., Groh C., Butcher N.J., Meinertzhagen I.A., Tavosanis G. (2009). Synaptic organization in the adult Drosophila mushroom body calyx. J. Comp. Neurol..

[83] Papadopoulou M., Cassenaer S., Nowotny T., Laurent G. (2011). Normalization for sparse encoding of odors by a wide-field interneuron. Science.

[84] Tanaka N.K., Tanimoto H., Ito K. (2008). Neuronal assemblies of the Drosophila mushroom body. J. Comp. Neurol..

[85] Liu X., Davis R.L. (2009). The GABAergic anterior paired lateral neuron suppresses and is suppressed by olfactory learning. Nat. Neurosci..

[86] Lei Z., Chen K., Li H., Liu H., Guo A. (2013). The GABA system regulates the sparse coding of odors in the mushroom bodies of Drosophila. Biochem. Biophys. Res. Commun..

[87] Liu X., Buchanan M.E., Han K.A., Davis R.L. (2009). The GABAA receptor RDL suppresses the conditioned stimulus pathway for olfactory learning. J. Neurosci..

[88] Christiansen F., Zube C., Andlauer T.F., Wichmann C., Fouquet W., Owald D., Mertel S., Leiss F., Tavosanis G., Luna A.J. (2011). Presynapses in Kenyon cell dendrites in the mushroom body calyx of Drosophila. J. Neurosci..

[89] Schwaerzel M., Monastirioti M., Scholz H., Friggi-Grelin F., Birman S., Heisenberg M. (2003). Dopamine and octopamine differentiate between aversive and appetitive olfactory memories in Drosophila. J. Neurosci..

[90] Liu C., Placais P.Y., Yamagata N., Pfeiffer B.D., Aso Y., Friedrich A.B., Siwanowicz I., Rubin G.M., Preat T., Tanimoto H. (2012). A subset of dopamine neurons signals reward for odour memory in Drosophila. Nature.

[91] Claridge-Chang A., Roorda R.D., Vrontou E., Sjulson L., Li H., Hirsh J., Miesenbock G. (2009). Writing memories with light-addressable reinforcement circuitry. Cell.

[92] Aso Y., Siwanowicz I., Bracker L., Ito K., Kitamoto T., Tanimoto H. (2010). Specific dopaminergic neurons for the formation of labile aversive memory. Curr. Biol..

[93] Aso Y., Herb A., Ogueta M., Siwanowicz I., Templier T., Friedrich A.B., Ito K., Scholz H., Tanimoto H. (2012). Three dopamine pathways induce aversive odor memories with different stability. PLoS Genet..

[94] Waddell S. (2013). Reinforcement signalling in Drosophila; dopamine does it all after all. Curr. Opin. Neurobiol..

[95] Sitaraman D., LaFerriere H., Birman S., Zars T. (2012). Serotonin is critical for rewarded olfactory short-term memory in Drosophila. J. Neurogenet..

[96] Qin H., Cressy M., Li W., Coravos J.S., Izzi S.A., Dubnau J. (2012). Gamma neurons mediate dopaminergic input during aversive olfactory memory formation in Drosophila. Curr. Biol..

[97] Wright G.A., Mustard J.A., Simcock N.K., Ross-Taylor A.A., McNicholas L.D., Popescu A., Marion-Poll F. (2010). Parallel reinforcement pathways for conditioned food aversions in the honeybee. Curr. Biol..

[98] Burke C.J., Waddell S. (2011). Remembering nutrient quality of sugar in Drosophila. Curr. Biol..

[99] Fujita M., Tanimura T. (2011). Drosophila evaluates and learns the nutritional value of sugars. Curr. Biol..

[100] Kim Y.C., Lee H.G., Han K.A. (2007). D1 dopamine receptor dDA1 is required in the mushroom body neurons for aversive and appetitive learning in Drosophila. J. Neurosci..

[101] Miyamoto T., Slone J., Song X., Amrein H. (2012). A fructose receptor functions as a nutrient sensor in the Drosophila brain. Cell.

[102] Dus M., Ai M., Suh G.S. (2013). Taste-independent nutrient selection is mediated by a brain-specific Na(+)/solute co-transporter in Drosophila. Nat. Neurosci..

[103] Quinn W.G., Dudai Y. (1976). Memory phases in Drosophila. Nature.

[104] Folkers E., Drain P., Quinn W.G. (1993). Radish, a Drosophila mutant deficient in consolidated memory. Proc. Natl. Acad. Sci. USA.

[105] Pagani M.R., Oishi K., Gelb B.D., Zhong Y. (2009). The phosphatase SHP2 regulates the spacing effect for long-term memory induction. Cell.

[106] Waddell S., Armstrong J.D., Kitamoto T., Kaiser K., Quinn W.G. (2000). The amnesiac gene product is expressed in two neurons in the Drosophila brain that are critical for memory. Cell.

[107] Keene A.C., Stratmann M., Keller A., Perrat P.N., Vosshall L.B., Waddell S. (2004). Diverse odor-conditioned memories require uniquely timed dorsal paired medial neuron output. Neuron.

[108] Yu D., Keene A.C., Srivatsan A., Waddell S., Davis R.L. (2005). Drosophila DPM neurons form a delayed and branch-specific memory trace after olfactory classical conditioning. Cell.

[109] Keene A.C., Krashes M.J., Leung B., Bernard J.A., Waddell S. (2006). Drosophila dorsal paired medial neurons provide a general mechanism for memory consolidation. Curr. Biol..

[110] Feany M.B., Quinn W.G. (1995). A neuropeptide gene defined by the Drosophila memory mutant amnesiac. Science.

[111] Tamura T., Chiang A.S., Ito N., Liu H.P., Horiuchi J., Tully T., Saitoe M. (2003). Aging specifically impairs amnesiac-dependent memory in Drosophila. Neuron.

[112] Cervantes-Sandoval I., Davis R.L. (2012). Distinct traces for appetitive versus aversive olfactory memories in DPM neurons of Drosophila. Curr. Biol..

[113] Lee P.T., Lin H.W., Chang Y.H., Fu T.F., Dubnau J., Hirsh J., Lee T., Chiang A.S. (2011). Serotonin-mushroom body circuit modulating the formation of anesthesia-resistant memory in Drosophila. Proc. Natl. Acad. Sci. USA.

[114] Pitman J.L., Huetteroth W., Burke C.J., Krashes M.J., Lai S.L., Lee T., Waddell S. (2011). A pair of inhibitory neurons are required to sustain labile memory in the Drosophila mushroom body. Curr. Biol..

[115] Placais P.Y., Trannoy S., Isabel G., Aso Y., Siwanowicz I., Belliart-Guerin G., Vernier P., Birman S., Tanimoto H., Preat T. (2012). Slow oscillations in two pairs of dopaminergic neurons gate long-term memory formation in Drosophila. Nat. Neurosci..

[116] Berry J.A., Cervantes-Sandoval I., Nicholas E.P., Davis R.L. (2012). Dopamine is required for learning and forgetting in Drosophila. Neuron.

[117] Krashes M.J., Keene A.C., Leung B., Armstrong J.D., Waddell S. (2007). Sequential use of mushroom body neuron subsets during drosophila odor memory processing. Neuron.

[118] Keene A.C., Waddell S. (2007). Drosophila olfactory memory: single genes to complex neural circuits. Nat. Rev. Neurosci..

[119] Chance F.S., Abbott L.F. (2000). Divisive inhibition in recurrent networks. Network.

[120] Wu C.L., Shih M.F., Lai J.S., Yang H.T., Turner G.C., Chen L., Chiang A.S. (2011). Heterotypic gap junctions between two neurons in the Drosophila brain are critical for memory. Curr. Biol..

[121] Huang C., Zheng X., Zhao H., Li M., Wang P., Xie Z., Wang L., Zhong Y. (2012). A permissive role of mushroom body alpha/beta core neurons in long-term memory consolidation in Drosophila. Curr. Biol..

[122] Rescorla R.A. (1973). Effect of US habituation following conditioning. J. Comp. Physiol. Psychol..

[123] Shuai Y., Lu B., Hu Y., Wang L., Sun K., Zhong Y. (2010). Forgetting is regulated through Rac activity in Drosophila. Cell.

[124] Folkers E., Waddell S., Quinn W.G. (2006). The Drosophila radish gene encodes a protein required for anesthesia-resistant memory. Proc. Natl. Acad. Sci. USA.

[125] Formstecher E., Aresta S., Collura V., Hamburger A., Meil A., Trehin A., Reverdy C., Betin V., Maire S., Brun C. (2005). Protein interaction mapping: a Drosophila case study. Genome Res..

[126] Blum A.L., Li W., Cressy M., Dubnau J. (2009). Short- and long-term memory in Drosophila require cAMP signaling in distinct neuron types. Curr. Biol..

[127] Trannoy S., Redt-Clouet C., Dura J.M., Preat T. (2011). Parallel processing of appetitive short- and long-term memories in Drosophila. Curr. Biol..

[128] Zars T., Fischer M., Schulz R., Heisenberg M. (2000). Localization of a short-term memory in Drosophila. Science.

[129] McGuire S.E., Le P.T., Davis R.L. (2001). The role of Drosophila mushroom body signaling in olfactory memory. Science.

[130] Akalal D.B., Wilson C.F., Zong L., Tanaka N.K., Ito K., Davis R.L. (2006). Roles for Drosophila mushroom body neurons in olfactory learning and memory. Learn Mem..

[131] Schwaerzel M., Heisenberg M., Zars T. (2002). Extinction antagonizes olfactory memory at the subcellular level. Neuron.

[132] Isabel G., Pascual A., Preat T. (2004). Exclusive consolidated memory phases in Drosophila. Science.

[133] Yu D., Akalal D.B., Davis R.L. (2006). Drosophila alpha/beta mushroom body neurons form a branch-specific, long-term cellular memory trace after spaced olfactory conditioning. Neuron.

[134] Akalal D.B., Yu D., Davis R.L. (2010). A late-phase, long-term memory trace forms in the gamma neurons of Drosophila mushroom bodies after olfactory classical conditioning. J. Neurosci..

[135] Pai T.P., Chen C.C., Lin H.H., Chin A.L., Lai J.S., Lee P.T., Tully T., Chiang A.S. (2013). Drosophila ORB protein in two mushroom body output neurons is necessary for long-term memory formation. Proc. Natl. Acad. Sci. USA.

[136] Perisse, E., Yin, Y., Lin, A.C., Lin, S., Huetteroth, W., and Waddell, S. Different Kenyon cell populations drive learned approach and avoidance in Drosophila. Neuron, *in press*. 10.1016/j.neuron.2013.07.045 PMC3765960 24012007

[137] Gerber B., Tanimoto H., Heisenberg M. (2004). An engram found? Evaluating the evidence from fruit flies. Curr. Opin. Neurobiol..

[138] Sejourne J., Placais P.Y., Aso Y., Siwanowicz I., Trannoy S., Thoma V., Tedjakumala S.R., Rubin G.M., Tchenio P., Ito K. (2011). Mushroom body efferent neurons responsible for aversive olfactory memory retrieval in Drosophila. Nat. Neurosci..

[139] Chen C.C., Wu J.K., Lin H.W., Pai T.P., Fu T.F., Wu C.L., Tully T., Chiang A.S. (2012). Visualizing long-term memory formation in two neurons of the Drosophila brain. Science.

[140] Shih H.W., Chiang A.S. (2011). Anatomical characterization of thermosensory AC neurons in the adult Drosophila brain. J. Neurogenet..

[141] Hirano Y., Masuda T., Naganos S., Matsuno M., Ueno K., Miyashita T., Horiuchi J., Saitoe M. (2013). Fasting launches CRTC to facilitate long-term memory formation in Drosophila. Science.

[142] Krashes M.J., DasGupta S., Vreede A., White B., Armstrong J.D., Waddell S. (2009). A neural circuit mechanism integrating motivational state with memory expression in Drosophila. Cell.

[143] Placais P.Y., Preat T. (2013). To favor survival under food shortage, the brain disables costly memory. Science.

[144] Roozendaal B., McGaugh J.L. (2011). Memory modulation. Behav. Neurosci..

[145] Kemenes I., O’Shea M., Benjamin P.R. (2011). Different circuit and monoamine mechanisms consolidate long-term memory in aversive and reward classical conditioning. Eur. J. Neurosci..

